# Seven tesla knee MRI T2*-mapping detects intrasubstance meniscus degeneration in patients with posterior root tears

**DOI:** 10.1093/radadv/umae005

**Published:** 2024-03-19

**Authors:** Abdul Wahed Kajabi, Štefan Zbýň, Jesse S Smith, Eisa Hedayati, Karsten Knutsen, Luke V Tollefson, Morgan Homan, Hasan Abbasguliyev, Takashi Takahashi, Gregor J Metzger, Robert F LaPrade, Jutta M Ellermann

**Affiliations:** Center for Magnetic Resonance Research, University of Minnesota, Minneapolis, MN, 55455, United States; Department of Radiology, University of Minnesota, Minneapolis, MN, 55455, United States; Center for Magnetic Resonance Research, University of Minnesota, Minneapolis, MN, 55455, United States; Department of Radiology, University of Minnesota, Minneapolis, MN, 55455, United States; Department of Biomedical Engineering, Lerner Research Institute, Cleveland Clinic, Cleveland, OH, 44196, United States; Center for Magnetic Resonance Research, University of Minnesota, Minneapolis, MN, 55455, United States; Department of Radiology, University of Minnesota, Minneapolis, MN, 55455, United States; Diagnostic Radiology, Oregon Health & Science University, Portland, OR, 97239, United States; Center for Magnetic Resonance Research, University of Minnesota, Minneapolis, MN, 55455, United States; Department of Radiology, University of Minnesota, Minneapolis, MN, 55455, United States; Center for Magnetic Resonance Research, University of Minnesota, Minneapolis, MN, 55455, United States; Department of Radiology, University of Minnesota, Minneapolis, MN, 55455, United States; Twin Cities Orthopedics, Edina, MN, 55435, United States; Twin Cities Orthopedics, Edina, MN, 55435, United States; Department of Diagnostic and Interventional Radiology, Ataturk University Research Hospital, Erzurum, 25240, Türkiye; Department of Radiology, University of Minnesota, Minneapolis, MN, 55455, United States; Center for Magnetic Resonance Research, University of Minnesota, Minneapolis, MN, 55455, United States; Twin Cities Orthopedics, Edina, MN, 55435, United States; Center for Magnetic Resonance Research, University of Minnesota, Minneapolis, MN, 55455, United States; Department of Radiology, University of Minnesota, Minneapolis, MN, 55455, United States

**Keywords:** ultra-high field MRI, 7T, T2*-mapping, meniscus, root tear

## Abstract

**Background:**

Medial meniscus root tears often lead to knee osteoarthritis. The extent of meniscal tissue changes beyond the localized root tear is unknown.

**Purpose:**

To evaluate if 7 Tesla 3D T2*-mapping can detect intrasubstance meniscal degeneration in patients with arthroscopically verified medial meniscus posterior root tears (MMPRTs), and assess if tissue changes extend beyond the immediate site of the posterior root tear detected on surface examination by arthroscopy.

**Methods:**

In this prospective study we acquired 7 T knee MRIs from patients with MMPRTs and asymptomatic controls. Using a linear mixed model, we compared T2* values between patients and controls, and across different meniscal regions. Patients underwent arthroscopic assessment before MMPRT repair. Changes in pain levels before and after repair were calculated using Knee Injury & Osteoarthritis Outcome Score (KOOS). Pain changes and meniscal extrusion were correlated with T2* using Pearson correlation (*r*).

**Results:**

Twenty patients (mean age 53 ± 8; 16 females) demonstrated significantly higher T2* values across the medial meniscus (anterior horn, posterior body and posterior horn: all *P *<* *.001; anterior body: *P *=* *.007), and lateral meniscus anterior (*P *=* *.024) and posterior (*P *<* *.001) horns when compared to the corresponding regions in ten matched controls (mean age 53 ± 12; 8 females). Elevated T2* values were inversely correlated with the change in pain levels before and after repair. All patients had medial meniscal extrusion of ≥2 mm. Arthroscopy did not reveal surface abnormalities in 70% of patients (14 out of 20).

**Conclusions:**

Elevated T2* values across both medial and lateral menisci indicate that degenerative changes in patients with MMPRTs extend beyond the immediate vicinity of the posterior root tear. This suggests more widespread meniscal degeneration, often undetected by surface examinations in arthroscopy.

**Abbreviations**

GRE = gradient-recalled echo, ICCs = intraclass correlation coefficients, KOOS = knee injury and osteoarthritis outcome score, MMPRTs = medial meniscus posterior; root tears, OA = osteoarthritis.

**Summary**

Seven Tesla MRI T2*-mapping of knees with medial meniscus posterior root tears detects significant intrasubstance degeneration in menisci that appeared largely normal during arthroscopy.

**Key Points**

- Patients with medial meniscus posterior root tears exhibited significantly elevated intrasubstance T2* values across both menisci extending far beyond the immediate tear site.
- These findings suggest widespread intrasubstance meniscus degeneration, frequently undetected by arthroscopic surface examination.
- This study introduces a clinically feasible method for meniscal tissue assessment using 3D T2*-mapping acquired in approximately 5 minutes, leveraging the high signal-to-noise ratio achievable at 7 Tesla.

## Introduction

Medial meniscus posterior root tears (MMPRTs) lead to meniscal extrusion, which is one of the strongest risk factors for the development and progression of knee osteoarthritis (OA).^1^^,^^2^ Accurate diagnosis and timely repair lead to improved patient outcomes.^3^ However, research suggests meniscal root repairs, which are now standard of care, may not reposition the meniscal tissue within the joint space, and postoperative meniscal extrusion often develops despite an intact repair site.^4^ Therefore, understanding the preoperative state of the meniscal tissue is crucial for orthopedic surgeons when planning treatment strategies, such as considering additional peripheral stabilization sutures to augment meniscal root repairs.

Arthroscopy is recognized as the gold standard to validate MRI findings. However, it only relies on visual inspection of the meniscal surface.

Standard clinical MRI protocols, even those at 3 T, may fall short in evaluating the quality of the meniscal tissue and the reparability of the meniscal root tear.^5^^,^^6^ Novel MRI methods for the detection of intra-meniscal tissue integrity have great potential for an improved understanding of meniscal degeneration and for the evaluation of treatment interventions, particularly in patients with MMPRTs. Recent quantitative MRI studies of menisci ex *vivo* have established the correlation of T2 and T2*-mapping with histologically verified degenerative processes, in particular, related to collagen density and organization.^7–9^ These histologic studies validated that elevated T2* values were significantly correlated with the loss of collagen fiber organization.^10^ These changes have implications on the quality of the meniscal tissue, its ability to undergo a successful repair, and can potentially lead to meniscal extrusion, even after a successful meniscal repair. To the best of our knowledge, *in vivo* 3D (three-dimensional) T2*-mapping of the menisci at 7 T in a clinically feasible acquisition time of about 5 minutes in patients with arthroscopically validated MMPRTs has not yet been reported.

The purpose of this study was to utilize 3D T2*-mapping, leveraging the signal-to-noise ratio and spatial resolution gains at 7 T to evaluate the intrasubstance meniscal degeneration in patients with arthroscopically verified MMPRTs when compared to matched controls and assess if the tissue changes extend beyond the immediate vicinity of the tear detected on arthroscopic examination.

## Materials and methods

### Study population

**Figure 1.**
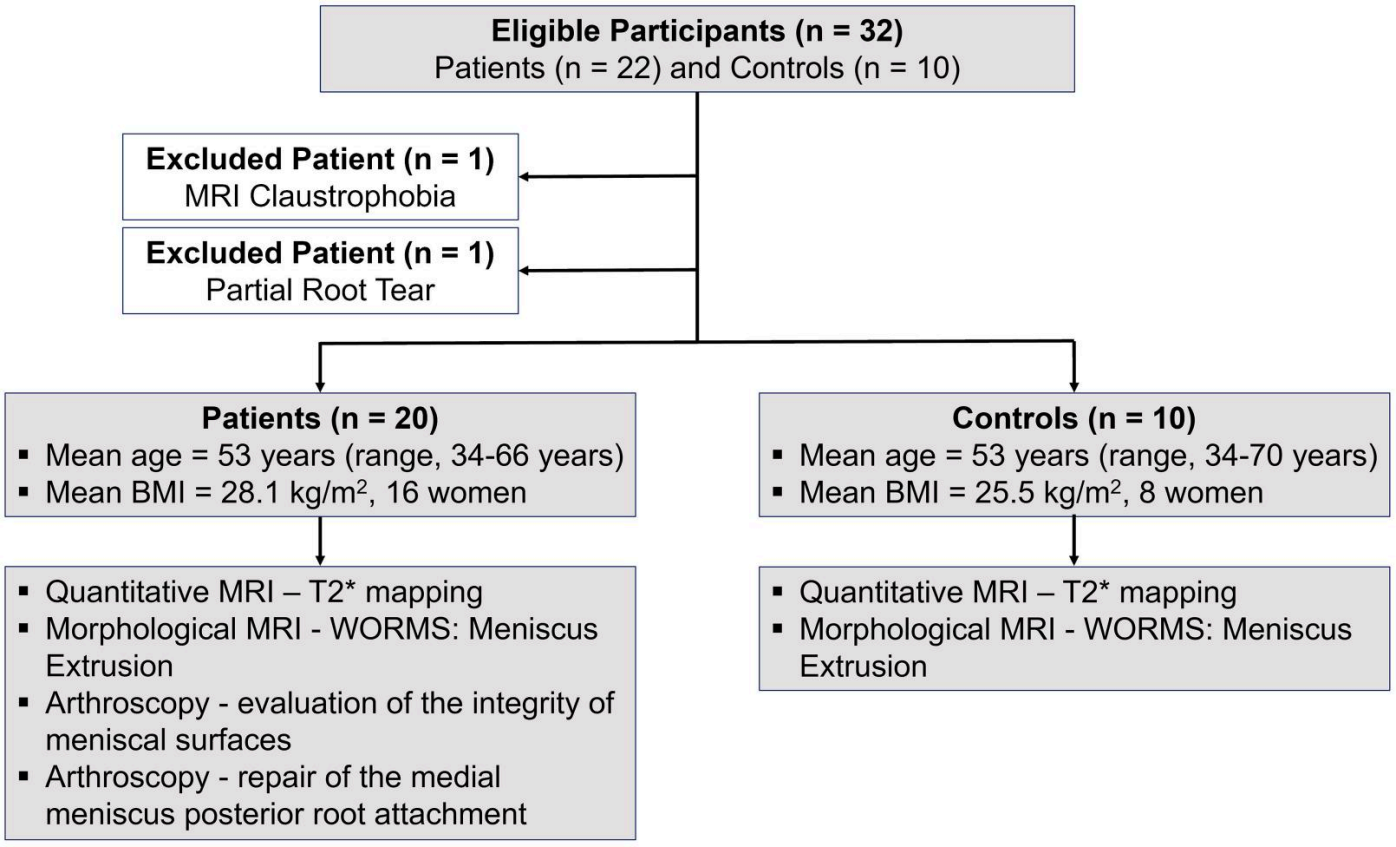
Patient flow, characteristics and evaluation metrics.

This prospective and Institutional Review Board approved study adheres to the Health Insurance Portability and Accountability Act (HIPAA). Informed consent was obtained from all participants who were consecutively enrolled from March 2021 to April 2023. Inclusion criteria were patients aged ≥ 18, suspected MMPRTs, and scheduled for knee arthroscopy. Also enrolled were age- and gender-matched asymptomatic controls without prior knee surgery (Table 1). Participants were excluded if they were pregnant, had MRI incompatible implants or had previous knee surgery (Figure 1).

**Table 1.** Demographic details of the study groups.

Group	Number of knees	Sex male/female	Mean age ± SD (age range) (years)	Mean BMI (kg/m^2^)	Root tear location medial/lateral	Symptoms (knee pain) Yes/No
Patients	20	4/16	53 **±** 8 (34-66)	28.1	20/0	20/0
Controls	10	2/8	53 **±** 12 (34-70)	25.5	n/a	0/10
Total	30	6/24	n/a	n/a	20/0	20/10

### Knee examination

All enrolled patients underwent a comprehensive knee examination by an experienced orthopedic surgeon (R.F.L., with >25 years of experience). Physical examination included (but was not limited to) inspection for joint-line tenderness, effusion, McMurray’s, and tibiofemoral stability tests.^11^ Moreover, the patients were asked to complete a questionnaire containing Knee Injury & Osteoarthritis Outcome Score (KOOS)^12^ for the assessment of knee pain they experienced before and six months after surgical repair.

### Arthroscopic evaluation

**Figure 2.**
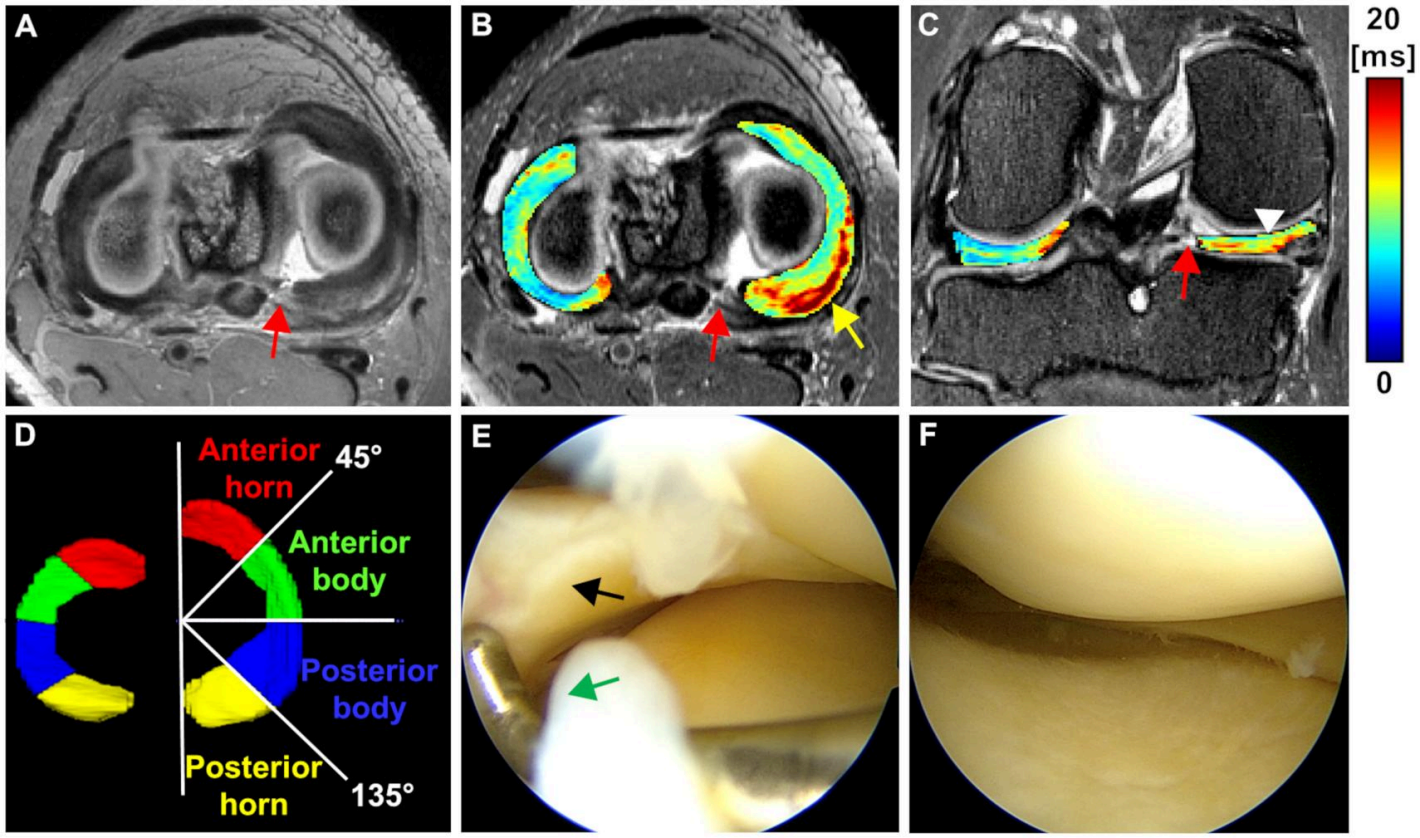
Representative MRI and arthroscopy images of a patient (age 57 years, female) with a posterior horn tear in the medial meniscus. A) Axial SPACE PD weighted image with arrow pointing to the root tear. B) Axial T2* map of the lateral and medial menisci overlaid on the axial SPACE T2 with fat suppression. Elevated T2* values are depicted in the posterior region proximal to the root tear (arrow pointing to the colormap). C) Coronal T2* map of the lateral and medial (arrowhead) menisci overlaid on the coronal SPACE T2 with fat suppression. D) 3D regional segmentation of the lateral and medial (regions annotated) menisci for quantitative analysis. E-F) preoperative arthroscopic images show the smooth surface of the medial meniscus. The arrows point to the medial meniscus root and the root attachment. The colour bar depicts T2* relaxation in milliseconds.

Arthroscopic evaluations of meniscal surface integrity were performed by the same orthopedic surgeon in patients prior to repair of the medial meniscus posterior root attachment (Figure 2E and F). Adopting the scoring system of Pauli et al.^13^ for the arthroscopic assessment of menisci surfaces, the integrity of the femoral and tibial meniscal surfaces and the inner rim of the medial and lateral menisci in each patient was categorized as: (i) smooth (grade = 0), (ii) slightly fibrillated or slightly undulating (grade = 1), (iii) moderate fibrillation or markedly undulating (grade = 2), and (iv) severe fibrillation or disruption (grade = 3).

### MRI protocol

All MRI data were acquired at 7 T Terra (Siemens, Erlangen, Germany) using a birdcage transmit/28-channel receive phased-array knee coil (Quality Electrodynamics, Mayfield Village, OH). The protocol included PD- and T2-weighted TSE, T2-weighted 3D SPACE sequences with and without fat suppression. For quantitative T2* mapping, a 3D multi-echo gradient-recalled echo (GRE) sequence was used with seven echo times (TEs, 3.1-21.4 ms). All sequence parameters are provided in Table 2.

**Table 2.** Knee MRI acquisition parameters for the morphological and T2*-mapping sequences.

Acquisition Parameter	Axial T_2_w FS TSE	Coronal T_2_w FS TSE	Sagittal PDw TSE	Sagittal T_2_w FS SPACE	Sagittal PDw SPACE	3D T2* mapping
Repetition time (ms)	6000	5750	3000	1500	1500	26
Echo time (ms)	42	42	32	22	58	3.1, 6.1, 9.2, 12.2, 15.3, 18.4, 21.4
Flip angle (degrees)	180	140	130	120	140	15
Pixel BW (Hz/pix)	320	265	560	680	765	360
Field of view (mm^2^)	129 × 129	139 × 139	139 × 139	128 × 128	125 × 125	130 × 130
Matrix size	384 × 307	496 × 372	496 × 347	320 × 320	272 × 272	300 × 300
In-plane resolution (mm^2^)	0.17 × 0.17	0.14 × 0.14	0.14 × 0.14	0.40 × 0.40	0.46 × 0.46	0.44 × 0.44
Slice thickness (mm)	3	2.5	2.5	0.45	0.46	1.0
Slice gap (mm)	0.45	0.95	0.95	n/a	n/a	n/a
Number of slices	32	31	31	256	240	120
Echo train length	8	8	8	60	38	7
GRAPPA/*CAIPIRINHA factor	2	2	3	*2 × 2	*2 × 2	3
Number of averages	1	1	1	1	1	1
Imaging time (min)	2:36	3:55	1:18	7:42	7:28	5:18

TSE = turbo spin echo; FS = fat suppression; PDw = proton density-weighted; SPACE = sampling perfection with application optimized contrast using different flip angle evolution

### Evaluation of the meniscal extrusion

Three musculoskeletal radiologists (J.M.E, T.T. and H.A. with 17, 9 and 4 years of experience, respectively) independently evaluated meniscal extrusion of patients and controls. The radiologists were blinded to T2*-mapping results. Meniscal extrusion was measured^14^ on coronal T2-weighted fat-suppressed TSE images using two vertical lines at the peripheral margins of the tibial plateau and the most peripheral aspect of the meniscal body on the image with the best visualization of the respective tibial eminence, as previously described.^14^ Extrusion was reported if the measurement was ≥2 mm. After independent evaluations, the radiologists reviewed all extrusion measurements they evaluated differently and reached a consensus for each case.

### 3D Segmentations and quantitative T2*-mapping

The 3D segmentation of all menisci was performed (J.S.S., 4 years of experience, and supervised by J.M.E., 17 years of experience) on T2*-weighted images in 3D orthogonal planes using ITK-SNAP (Figure 2). The 3D SPACE images were referenced to accurately identify the meniscus. Menisci were segmented from just 1 mm distal to the anterior root attachment to about 1 pixel (0.44 mm) proximal to the posterior root tear. Control menisci segmentation mirrored this, extending from 1 mm distal of the anterior root to 1 mm proximal to the posterior root. Segmented menisci were subsequently divided into four regions based on angles relative to the line connecting anterior and posterior horns: (i) anterior horn (0°-45°), (ii) anterior body (45°-90°), (iii) posterior body (90°-135°), and (iv) posterior horn (135°-180°) (Figure 2D).

T2*-mapping was calculated by fitting a mono-exponential signal decay to the multi-echo T2* data with a two-parametric non-linear model using a least-squares fitting routine in MATLAB (MathWorks). The root mean square error (RMSE), normalized to the estimated signal intensity at TE of 0 ms was calculated to evaluate the fitting accuracy. Pixel count was used to calculate the volume of each meniscus. Median T2* values and the corresponding RMSE were measured in four meniscal regions (Figure 2D).

### T2*-mapping reproducibility analysis

To determine T2*-mapping reproducibility, four additional asymptomatic controls were imaged using the same MRI protocol and were acquired twice for each participant (scan-rescan) after repositioning the knee before each scan. Menisci of each participant from each scan were segmented by the same evaluator (J.S.S., 4 years of experience) using the same approach as in the torn meniscal root patients (Figure 2D).

### Statistical analysis

Normality of the T2* data distribution was confirmed by performing normality tests using Kolmogorov-Smirnov and Shapiro-Wilk tests. Comparisons were performed using a linear mixed effect model for meniscus volume and the T2* values between meniscal regions in patients and corresponding regions in the controls. This model was used to take into account the subject-level random effects,^15^ including age, gender, and body mass index (BMI) as covariates. The *P*-values of between-region comparisons were adjusted for pairwise comparisons between the four regions using Dunn-Bonferroni methods, separately for the medial and lateral meniscus of patients, and medial and lateral menisci of controls. For correlations, Pearson correlation (*r*) was used to test the correlation of T2* values with meniscal extrusion and pain scores.^12^

The scan-rescan reproducibility was analyzed using Bland-Altman plots^16^ and two-way intraclass correlation coefficients (ICCs) of absolute agreement of the repeated measurements for each analyzed region. One-sample *t*-test was used to evaluate the difference between the two measurements.

A *P*-value of less than .05 was considered as statistically significant. Statistical analyses were calculated using SPSS (version 28, IBM Corp, Armonk, NY).

## Results

### Participant characteristics

Of 22 patients, one claustrophobic patient and one patient with a partial root tear were excluded, and 20 patients (mean age, 53 years ± 8 [standard deviation]; range: 34-66 years; mean BMI, 28.1 kg/m^2^; 16 women) that underwent 7 T MRI with an arthroscopic diagnosis of a complete radial root tear in the posterior horn of the medial meniscus were included in the analysis (Table 1, Figure 1). Additionally, 10 gender-, age- and BMI-matched asymptomatic controls (mean age, 53 years ± 12; age range: 34-70 years; mean BMI, 25.5 kg/m^2^, 8 women) (Table 1) were scanned using the same MRI protocol.

### Knee examination and arthroscopic evaluation

Of 20 patients included in the analysis, 11 of them reported their pain scores^12^ before and six months after their surgical repair. The pain score difference reported by the patients before and after surgical repair revealed that 10 patients had higher KOOS pain scores, describing reduced pain six months after surgery. One patient did not report a difference between pre- and post-repair pain scores.

Arthroscopy confirmed MMPRTs in all patients and revealed low-grade (Pauli 1-2) meniscal surface changes in six of 20 patients (two patients: grade 2, four patients: grade 1). The medial meniscus surface changes were found on the femoral side (one patient: grade 1), tibial side (two patients: grade 1), and inner border (two patients: grade 1). The lateral meniscus showed changes on the femoral side (two patients: grade 2), tibial side (one patient: grade 1, one patient: grade 2), and inner border (four patients: grade 1). No visible abnormalities were observed in the remaining 14 patients.

### Meniscus extrusion

All 21 patients had medial meniscal extrusion between 2-5 mm. No lateral menisci or control group menisci met the criteria for meniscal extrusion.^14^

### Quantitative T2* evaluation

**Figure 3.**
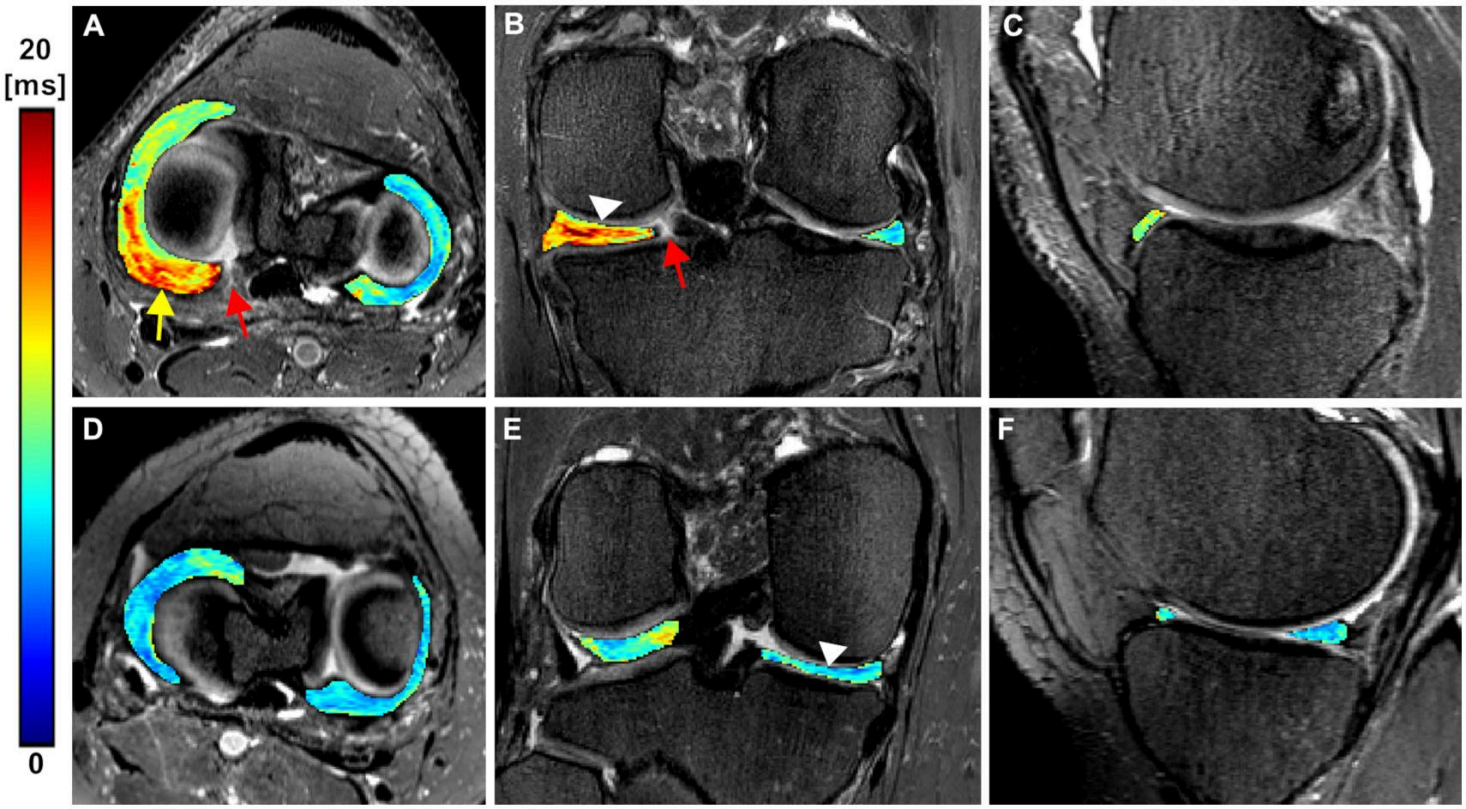
Representative 3D T2* maps overlaid on 3D SPACE fat-suppressed images of the medial and the lateral menisci of a patient (first row: A-C, age 62, male) with posterior horn root tear (arrow in B) in the medial meniscus and an asymptomatic control (second row: D-F, age 46, female). A, B) The segmented T2* maps show increased T2* values (arrowhead in B) in the medial meniscus of the patient particularly in the posterior body and posterior horn, as compared to the control (arrowhead in E). The colour bar depicts T2* relaxation in milliseconds.

**Figure 4.**
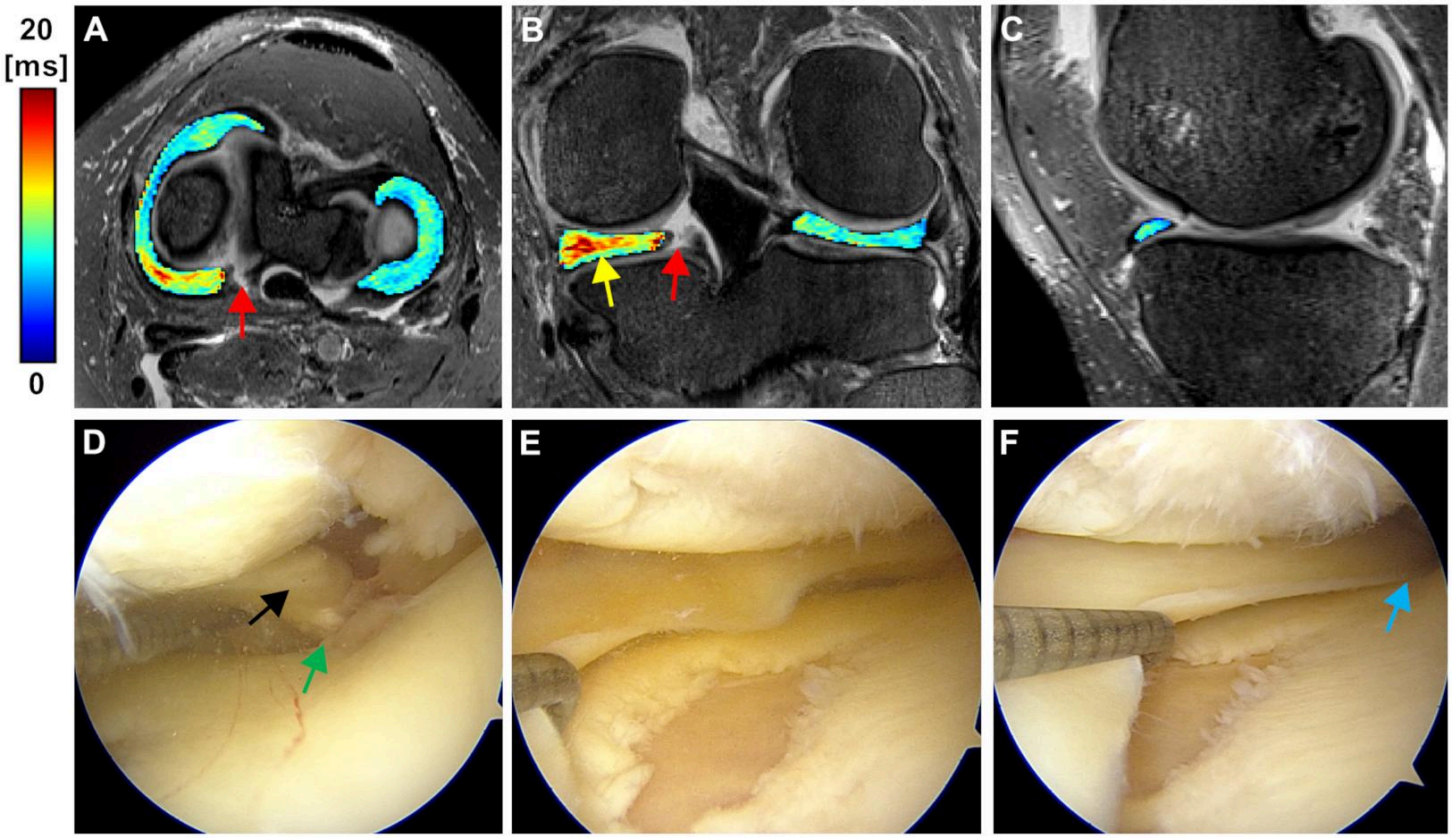
Representative MRI and arthroscopy images of a patient (age 57, female) with no arthroscopic findings (grade 0). A-C) 3D T2* maps overlaid on 3D SPACE fat-suppressed images of the medial and the lateral menisci with posterior horn root tear (arrow in A) in the medial meniscus. The segmented T2* maps show increased T2* values (arrow in B pointing to the colormap), particularly in the posterior region of the medial meniscus. The colour bar depicts T2* relaxation in milliseconds. D, E) Pre-operative arthroscopy images of the patient showing the medial meniscus root and the root attachment (arrows in D), and no signs of fibrillation of the medial meniscus. F) Post-operative arthroscopy image of the patient depicting the root repair (arrow).

**Figure 5.**
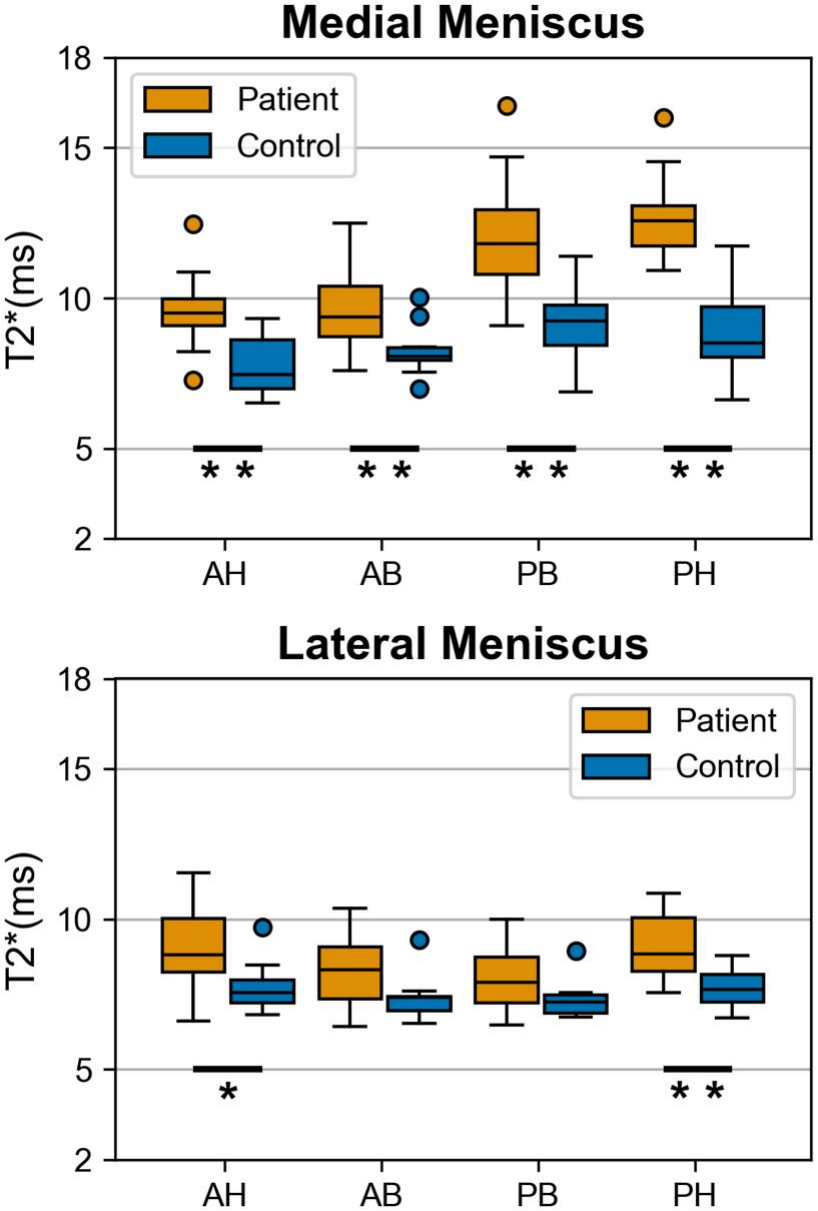
Boxplots of the T2* values from meniscus anterior horn (AH), anterior body (AB), posterior body (PB) and posterior horn (PH) of 20 patients with a posterior horn tear in the medial meniscus and closely age- and gender-matched 10 asymptomatic controls. The T2* relaxation times in patients were significantly longer (*P < .05; **P < .001) compared to the control group in all analyzed regions of the medial meniscus and the anterior and posterior horns of the lateral meniscus. The horizontal lines inside the boxes represent the mean T2*, and the upper and lower whiskers extend to the maximum and minimum T2* in each region, respectively. The boxes in the boxplots represent the interquartile range between the first quartile (ie, 25^th^ percentile) and third quartile (ie, 75^th^ percentile).

The T2* relaxation times of the medial meniscus of patients were significantly longer in the anterior horn, anterior body, posterior body, and posterior horn (all *P-values* < .001 except anterior body: *P *=* *.007) comparing to the corresponding regions relaxation times in the matched controls (Table 3, Figures 2-5). In the lateral meniscus, the T2* values were significantly longer in the anterior horn (*P *=* *.024) and posterior horn (*P *<* *.001), but not in the anterior body (*P *=* *.065) and posterior body (*P *=* *.061) of the patients when compared to the corresponding regions in controls (Table 3, Figure 5).

**Table 3.** Mean (standard deviation, SD) of the median T2* relaxation times (ms) for the analyzed regions and the total volume (number of pixels) for the lateral and medial menisci of the patients and the controls.

Regions	Patient T2* (ms)	Control T2* (ms)	*P*-values	Patient Volume (pixels)	Control Volume (pixels)	*P*-values
*Medial meniscus*						
Anterior horn	9.57 (1.05)	7.74 (0.98)	<**.001**	10395 (2310)	7104 (1790)	<**.001**
Anterior body	9.53 (1.24)	8.25 (0.88)	**=.007**			
Posterior body	11.95 (1.88)	9.19 (1.35)	<**.001**			
Posterior horn	12.68 (1.31)	8.82 (1.46)	<**.001**			
*Lateral meniscus*						
Anterior horn	8.89 (1.37)	7.75 (0.87)	=**.024**	7426 (3196)	4870 (1233)	**.022**
Anterior body	8.17 (1.18)	7.38 (0.77)	=.065			
Posterior body	8.01 (1.01)	7.31 (0.65)	=.061			
Posterior horn	9.06 (1.00)	7.70 (0.67)	<**.001**			

Regional comparisons in the medial meniscus of patients showed significantly higher T2* values in the posterior body (*P *<* *.001) and posterior horn (*P *<* *.001) when compared to the anterior horn and anterior body. No significant differences were found between the anterior horn and anterior body (*P *=* *1.000) or between the posterior body and posterior horn (*P *=* *.113). In the lateral meniscus of the patients, T2* values were significantly longer in the anterior and posterior horns when compared to the anterior body (*P *=* *.008, *P *<* *.001) and posterior body (*P *<* *.001, *P *<* *.001), respectively.

In the control group medial meniscus, T2* values were also significantly longer in the posterior horn (*P *=* *.002) and posterior body (*P *<* *.001) when compared to the anterior horn. Moreover, the posterior body had significantly longer T2* (*P *=* *.008) compared to the anterior body. No significant differences were found between the regions of the lateral meniscus of the controls.

Volumes of the medial (*P *<* *.001) and the lateral (*P *=* *.022) menisci were significantly larger in patients than in controls (Table 3).

### Correlations

Moderate negative correlations (ranging between *r *=* *−0.440 and *r *=* *−0.585) were found between the increased T2* values in different regions of the medial meniscus and the difference in pain scores reported by the patients at baseline and six months after repair (Table 4). In other words, lower pre-repair T2* values were associated with decreased pain after the meniscal repair. No correlation was found between the medial meniscus extrusion measurements versus volume (*r *=* *−0.065), and extrusion versus T2* values (*r *=* *0.108).

**Table 4.** Pearson correlation coefficients (*r*) and 95% confidence interval (CI) between medial meniscus T2* values and pain score difference reported by the patients before and six months after root repair.

Regions	Patient count	Correlation coefficient (*r*)	Lower CI	Upper CI	*P*-values
*Medial meniscus*					
Anterior horn	11	−0.556	−0.867	−0.066	.076
Anterior body	11	−0.440	−0.823	0.217	.175
Posterior body	11	−0.585	−0.877	0.023	.059
Posterior horn	11	−0.476	−0.837	0.174	.139

### T2* reproducibility evaluations

**Figure 6.**
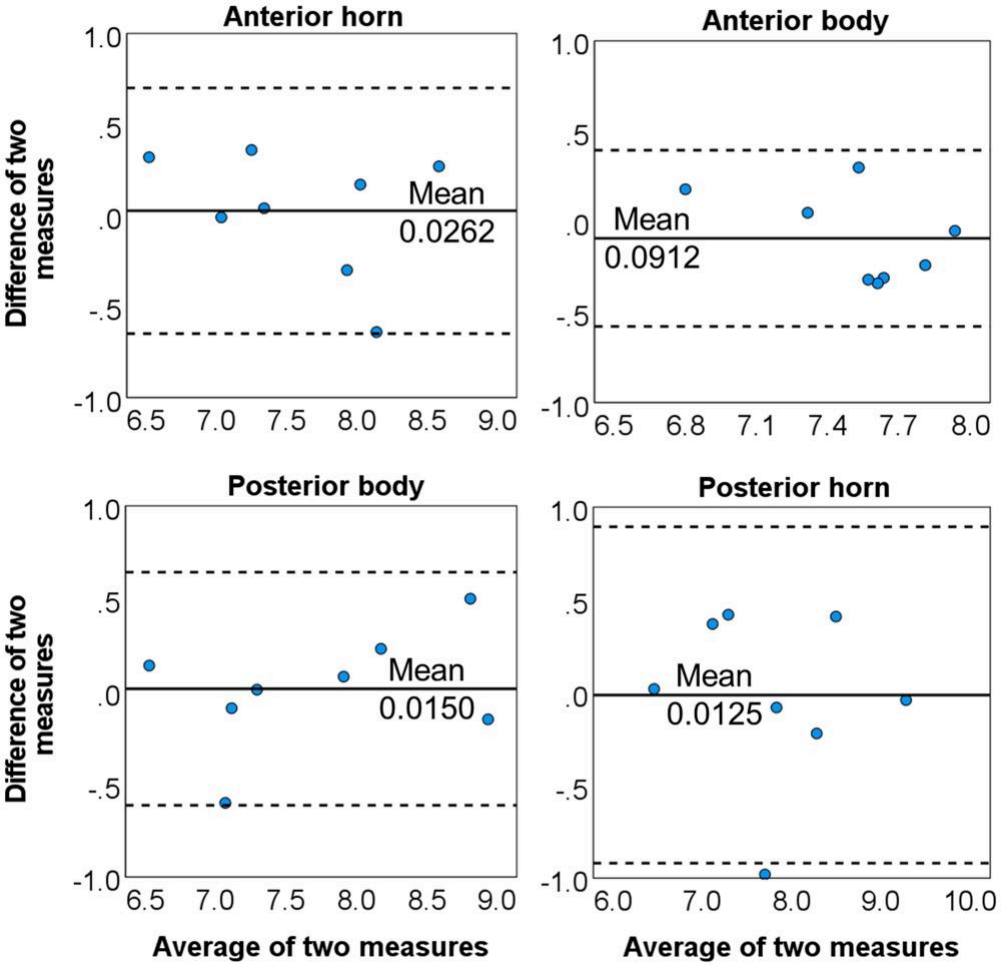
The Bland-Altman plots of the analyzed meniscal regions for the measurements acquired from scan and re-scan of the same participants. The solid line is the mean difference *d*, and the dotted lines are *d −* 1.96 *SD* (lower) and *d *+* *1.96 *SD* (upper). (SD = standard deviation).

The correlation between the scan and rescan T2* measurements in each meniscal region was good to excellent^17^ (anterior horn: ICC* *=* *0.94, 95% CI* *=* *0.71-0.99; anterior body: ICC = 0.87, 95% CI = 0.42-0.97; posterior body: ICC* *=* *0.97, 95% CI* *=* *0.85-0.99; posterior horn: ICC = 0.94, 95% CI = 0.69-0.99). No significant differences were found between the scan and rescan measurements in the four meniscal regions (all *P-values* ≥ .334). The consistency between the two measurements is depicted with Band-Altman plots^16^ (Figure 6).

## Discussion

This study showed that increased T2* values, reflective of meniscal degeneration,^7^^,^^8^ were not confined to the immediate area of the posterior root tear, but affected the entire medial meniscus, as well as the anterior and the posterior horns of the lateral meniscus. In addition, meniscal volume was significantly larger in the study patients than in controls. Increased T2* values were observed in larger meniscal volumes supporting the idea that changes in collagen fiber binding and hydration contribute to meniscus degeneration.

In this study, all 7 T MRI MMPRT diagnoses were confirmed arthroscopically. Quantitative T2*-mapping identified meniscal intrasubstance degeneration in the patients that were not apparent during arthroscopic repair, both near the MMPRTs and in distant regions. The correlation between T2* values and the meniscal collagen network has been established in previous ex vivo MRI studies and in vitro gold standard histology. Hager et al.^18^ demonstrated that T2*-mapping is sensitive to changes in the density and orientation of meniscal collagen fibers. Previous studies found T2*-mapping capable of detecting subclinical meniscal degeneration and torn menisci.^9^^,^^10^ Elevated T2* values were significantly correlated with the loss of collagen fiber organization as determined by histopathology.^10^ Pain improved after surgical repair in 10 out of 11 patients with available scores and was moderately correlated with T2*.

Meniscal extrusion leads to an inability to restore normal joint contact forces which can lead to OA.^19^ Meniscal extrusion has been reported in up to 38.8% of root repairs.^4^ In MMPRT repair patients, extrusion is common postoperatively, and cartilage scores decline over 2 to 5 years, even with maintained functional outcomes.^20^ This has significant clinical implications, since the failure rate of meniscal root repairs are high, reported to be at 20-24%.^21^^,^^22^ Our findings have important implications in filling the current gap of knowledge on the failure of meniscal root repair. The study’s quantitative findings of increased T2* values not just near the MMPRTs but also across a larger area of the medial and even the lateral meniscus, along with greater meniscus volume, align with histological research linking such patterns to disrupted collagen fibers.^10^^,^^18^ This loss of collagen fiber organization can lead to meniscal tissue laxity, prone to extrusion, loss of function, and potentially compromising the effectiveness of meniscal repair surgery.

Clinical imaging based on T2- or PD-weighted contrast does not quantitatively capture the intra-meniscal damage. Quantitative characterization of T2* using a multi-echo GRE acquisition adds to the utility of MRI to aid in both diagnosis and follow-up of menisci. Due to the range of T2* values (6-15ms) present in healthy and diseased tissue, standard Cartesian GRE acquisitions with the acquired TEs were sufficient to capture the contrast of interest. Furthermore, MRI sequences similar to the ones used in this study are available on all clinical MRI systems, improving the potential for adoption by the radiological community.

FDA approval of 7 T knee imaging enables high-resolution, clinically practical assessment of meniscal quality beyond 3 T capabilities. 7 T MRI’s superior signal and contrast to noise ratios^23^^,^^24^ enhance image quality, allowing for higher spatial resolution and/or faster acquisition. Our study collected a full 3D dataset of the knee joint in about 5 minutes, a four-fold acceleration with a 3-fold higher spatial resolution when compared to a similar prior study.^9^ This represents a fundamental advancement in applying 7 Tesla MRI in a clinical setting. Additionally, 7 T provides superior diagnostic accuracy for detecting meniscal damage over 3 T, utilizing its higher resolution capabilities.^25^

Our study had some limitations. First, the control group was small (n = 10), which reduced statistical power and resulted in an asymmetric comparison with the patient population. However, a Linear Mixed Model used for the statistical analysis accounted for the asymmetric comparison.^15^ Also, T2*-mapping is sensitive to susceptibility changes resulting from tissue interfaces and potential partial volume effect from cartilage and synovial fluid. To mitigate this potential variability, a 7 T high-resolution 3D-acquisition scheme was employed, menisci were carefully segmented to avoid inclusion of cartilage and synovial fluid, and median, rather than mean T2* values are reported. Furthermore, the reproducibility of T2* measurements in meniscal regions was found to be good to excellent. The T2* results in this study were not correlated with pathology as this was an *in vivo* study of patients who underwent meniscal repair. Tissue quality was discussed based on prior histological studies correlating T2* with collagen fiber organization.^7–9^ The study utilized a 7 T MRI, which is less commonly available than 3 T. However, the adoption of clinical 7 T MRI scanners has outpaced those designated solely for research, with numerous institutions now acquiring a second clinical 7 T system.

In conclusion, patients with arthroscopically confirmed MMPRTs exhibited increased T2* values across both medial and lateral menisci indicating that degenerative changes in patients with MMPRTs extend beyond the immediate vicinity of the posterior root tear. This suggests more widespread meniscal degeneration, often undetected by surface examinations in arthroscopy and may have clinical implications on the potential success of a meniscal root repair.

## Supplementary Material

umae005_Supplementary_Data

## Data Availability

The original contributions presented in the study are included in the article. Further inquiries can be directed to the corresponding author.
